# Identifying salient behavioral health access and social determinant of health factors among a Latino population with uncontrolled diabetes living on the U.S.-Mexico border

**DOI:** 10.3389/fpubh.2026.1808640

**Published:** 2026-08-05

**Authors:** Lisa A. Mitchell-Bennett, MinJae Lee, Zihan Yang, Natalia I. Heredia, Juliana Z. Lopez, Maria E. Zolezzi, Paul G. Yeh, Joseph H. Conroy, Joseph B. McCormick, Belinda M. Reininger

**Affiliations:** 1 Brownsville Regional Campus, The University of Texas Health Science Center at Houston School of Public Health, Brownsville, TX, United States; 2 Division of Clinical and Translational Sciences, McGovern Medical School, The University of Texas Health Science Center at Houston, Houston, TX, United States; 3 Department of Biostatistics and Data Science, The University of Texas Health Science Center at Houston, Houston, TX, United States; 4 Department of Health Promotion & Behavioral Science, The University of Texas Health Science Center at Houston, Houston, TX, United States; 5 Department of Kinesiology, Rice University, Houston, TX, United States

**Keywords:** behavioral health access, diabetes, factor analysis, Latino, social determinants of health, U.S.-Mexico border

## Abstract

**Background:**

Valid measures of behavioral health access and social determinants of health (SDOH) can inform health promotion, programming, and strategies to address identified gaps. Existing SDOH screening tools and domains were not developed using data from low-income Latino populations or community-based interventions, which may limit their relevance for these purposes.

**Aim/Objectives:**

This study sought to identify salient constructs of behavioral health and SDOH based on factor analysis among a low-income, underinsured Latino population with type 2 diabetes.

**Methodology:**

We gathered data from 6,735 Latino participants from two Texas-Mexico border counties enrolled in a multi-year diabetes management intervention to evaluate associations between observed behavioral health access and SDOH variables. We characterized common themes using factor analysis.

**Results:**

We found one independent summary factor related to behavioral health access and four factors related to SDOH: 1. Insurance and health care barriers; 2. Major stress from lack of medical provider and resources; 3. Urgent financial or social hardship; and 4. Transportation barriers.

**Conclusion:**

The results inform a set of measures across multiple domains unburdened with multicollinearity and aligned with previous research on SDOH. The findings provide deep insight for strategies to address SDOH gaps using measures tailored to specific populations in clinical and community settings. Use of these measures could help better identify and intervene on the unmet needs that negatively impact health outcomes.

## Introduction

It is now widely understood that social determinants of health (SDOH), such as education, transportation, housing, employment, health insurance and food security, among other non-clinical drivers, influence physical and mental health outcomes across various populations (1–6). According to the World Health Organization (WHO), when there is unequal distribution of SDOH, it leads to health disparities, which are unfair, avoidable, and lead to differences in health status between social groups (7). While positive SDOH characterize protective influences on health (4), adverse SDOH characterize deficit social conditions in populations, leading to a disproportionate burden of poor health outcomes (8). Adverse SDOH, especially poverty, can detrimentally impact weight, sedentary behavior and metabolic syndrome and are associated with increased prevalence of both non-communicable and infectious diseases (2, 9–12). For example, area-level poverty is the strongest predictor of obesity (13, 14). Lack of health insurance also impacts health outcomes negatively, since the uninsured person receives less access to healthcare than their insured counterparts (15, 16). In sum, at its core, SDOH captures access to power, resources and money in communities and populations and can therefore drive health inequities (3).

While there has been increasing attention to SDOH over the last two decades, there is a lack of consistent, relevant and culturally-tailored screening approaches for SDOH that account for marginalized populations' experiences and consider multiple SDOH needs (9). Health care providers face barriers to screening and referral, such as discomfort discussing social needs and limited time due to competing priorities (4, 17). When SDOH screening occurs, however, it can result in cost-effective and comprehensive treatment of conditions that unequally burden certain populations. To facilitate SDOH interventions, however, we need appropriate measures that capture relevant needs (4, 18–21).

Efforts to standardize SDOH measurement tools and compare results across studies have been limited by the lack of consensus regarding terms and definitions (19–21). Some more prominent strategies to establish uniform SDOH screening measures include several initiatives including: The Gravity Project, a national public collaborative developing consensus-based data standards for SDOH measurement; the protocol for responding to & assessing patients' assets, risks & experiences (PRAPARE), SDOH screening tools developed for community health centers; and the U.S. Center for Medicaid's Accountable Health Communities Health-Related Social Needs Screening Tool (AHC) (22–24). Although consistency in measurement is important for comparing assessments and interventions across populations, studies in underrepresented populations remain necessary to better understand how SDOH may differ across settings and influence health outcomes.

Balancing the need for consistency in measurement is the need for tools to examine relevant SDOH for diverse populations. Understanding the relevance of specific drivers and barriers for a specific group can strengthen the strategies implemented to target disease management interventions and improve health (25, 26). A 2024 scoping review of SDOH screening and interventions found diverse approaches to assessing and addressing SDOH and an increase in home-grown screening tools tailored to specific patient populations and healthcare settings, demonstrating a need for programmatic-based tools (5, 27, 28). Populations that remain underrepresented in the literature, such as Latino and low-income uninsured populations, may particularly benefit from approaches tailored to their specific needs (29).

Programmatic service databases are an untapped source of important SDOH needs data in populations underrepresented in the research/literature. However, because these databases contain numerous correlated measures, reducing them to fewer meaningful domains improves interpretability, minimizes multicollinearity, and facilitates the examination of their associations with health outcomes. We used service delivery measures collected over time as part of a diabetes management intervention, rather than a one-time standardized SDOH screening tool. This allowed us to take an inductive approach and identify domains derived from participants' real-life experiences (5). The purpose of this study was to identify salient constructs of behavioral health and SDOH based on factor analysis among a low-income, underinsured Latino population with type 2 diabetes.

## Materials and methods

### Study design and setting

This study uses factor analysis to describe variability in observed behavioral health access and SDOH variables using their correlation structure, and to determine salient underlying dimensions (domains) to reduce multicollinearity. This work was conducted with a Latino population living with uncontrolled type 2 diabetes in a U.S.-Mexico border setting.

### Study population

We included data from 6735 Latino (predominantly Mexican American) participants drawn from clinical and community settings in two U.S.-Mexico border counties in Texas, Cameron and Hidalgo, enrolled in a multi-year intervention called “Salud y Vida” (SyV), between 2013 and 2020 (See additional file: “Participant Inclusion”). The SyV intervention was developed in 2012 to address the high rates of uncontrolled type 2 diabetes mellitus (T2DM) among Latino residents at the southernmost Texas border. The SyV program was tailored to assess and address the SDOH barriers faced by many people in the region. SyV uses the expanded chronic care management model featuring clinic and community partnerships and Community Health Worker (CHW) services delivered to the home (30, 31). Clinics, CHWs, social workers and community-based organizations work in tandem to assess and address SDOH, leading to improved T2DM control. Participants were enrolled in the program from safety-net clinics serving a primarily low-income, Latino population with uncontrolled T2DM and comorbidities like hypertension. Clinical measures such as HbA1c, blood pressure, and body mass index (BMI) were collected over time, and SDOH needs were assessed by CHWs and social workers during regular home visits to participants. The program model, outcomes, and population have been previously published (31, 32).

### Ethical considerations

The study was approved by the Committee for the Protection of Human Subjects (IRB# HSC-SPH-13-0532). Data for this study were accessed on May 25, 2023. For purposes of administering the ongoing SyV program, some authors have access to information in the program database that could identify individual participants; however, a separate research database was created for this study that de-identified all participant data for analysis. At time of enrollment in Salud y Vida, all participants provided written consent to participate in the intervention and to share information for care coordination and research.

### Measurement parameters

The measures for this study were captured in an intervention case management database used to track service delivery and implement a chronic care management program under a Centers for Medicare & Medicaid Services waiver program (33). This waiver encouraged innovative service delivery to achieve high performance; therefore, as the chronic care management program unfolded over its decade-long implementation, the need for and ability to address behavioral health access and adverse SDOH evolved. While not considered an SDOH, we included items related to behavioral health access needs (referrals and screening) together with SDOH needs, because Latinos are generally disproportionately at risk for not receiving mental health treatment (34). In this population, access to behavioral health screening and services is often negatively impacted by SDOH barriers and compounded by high stigma around mental health (34).

### Assessment parametrics (data sources of behavioral health access and SDOH)

We included 39 variables collected through program enrollment, health services and referrals provided throughout the course of program delivery and screenings conducted by program staff (Supplementary Table 1). These included a) demographic variables like education, insurance, employment status, preferred language and health literacy b) service delivery variables that included transportation, food delivery, housing and utility assistance, compassion funding for medications and medical visits, and referrals to social services and behavioral health, c) screening metrics that included validated tools such as PHQ-9 and non-validated tools that assessed key life stressors and barriers to diabetes management and medication adherence. To assess life stressors, participants were asked the question “What are the major stressors in your life?” with response options including: financial, financial resources for diabetes care, bills, employment related, general lack of resources, lack of medical resources, housing, unable to work, family in Mexico, unemployment, food, home safety, disability, depression, family member's death, family members health, family member's substance abuse, no primary care physician, general stress, justice system, family distant. This measure aligns with the behaviors associated with diabetes control from the American Association of Diabetes Educators' 7 Self-care Behaviors of Healthy Coping (35). All information was recorded in the program database. Variables are a mix of categorical, numeric, and binary (Supplementary Table 1). We compared the SyV behavioral health access and SDOH variables with SDOH definitions and frameworks found in the literature for similarities and gaps.

### Statistical analysis

Data from 6735 Latino participants from two Texas-Mexico border counties were included in statistical analysis (Supplementary Diagram 1). We investigated associations between observed behavioral health access and SDOH variables and characterized their common themes (i.e., domains/factors) using factor analysis, which yields summary factors in descending order of importance based on the proportion of variance in the observed data accounted for by each factor. All 39 behavioral health access and SDOH variables were retained in the analysis. Prior to factor analysis, variable distributions and response frequencies were reviewed to assess sparsity and variability. Although several variables had relatively low prevalence, none demonstrated a complete or near-complete lack of variability, warranting exclusion.

To determine the optimal number of factors, we considered multiple criteria, including eigenvalues greater than 1.0 (Kaiser criterion), inspection of the scree plot, interpretability of the factor structure, and parsimony of the resulting domains. The final five-factor solution was selected because it provided conceptually meaningful and clinically interpretable domains while minimizing overlap among variables and preserving adequately explained variance. Behavioral health access, and SDOH variables were considered meaningful contributors to each summary factor if their factor loadings were 0.40 or higher, consistent with commonly used conventions in exploratory factor analysis and the psychometric literature (36). This threshold was selected to balance interpretability with inclusion of clinically relevant variables. Most behavioral health access and SDOH variables included in the analysis were binary indicators derived from programmatic assessments and service documentation.

Although exploratory factor analysis is traditionally developed for continuous variables, it has also been widely applied in large-sample health services and survey research involving binary or ordinal variables to identify latent constructs and reduce dimensionality. Given the large sample size and the exploratory objective of identifying clinically meaningful domains, factor analysis was deemed appropriate. To further evaluate the robustness of the identified factor structure for categorical data, we additionally performed K-modes clustering (37), an unsupervised clustering approach specifically designed for categorical variables.

We further evaluated the associations between demographic and other baseline characteristics and the identified SDOH factors using the Chi-square test. All analyses were conducted using SAS 9.4 software (SAS Institute Inc., Cary, NC) and R (R Core Team) at a significance level of 0.05.

## Results

**Figure 1:**
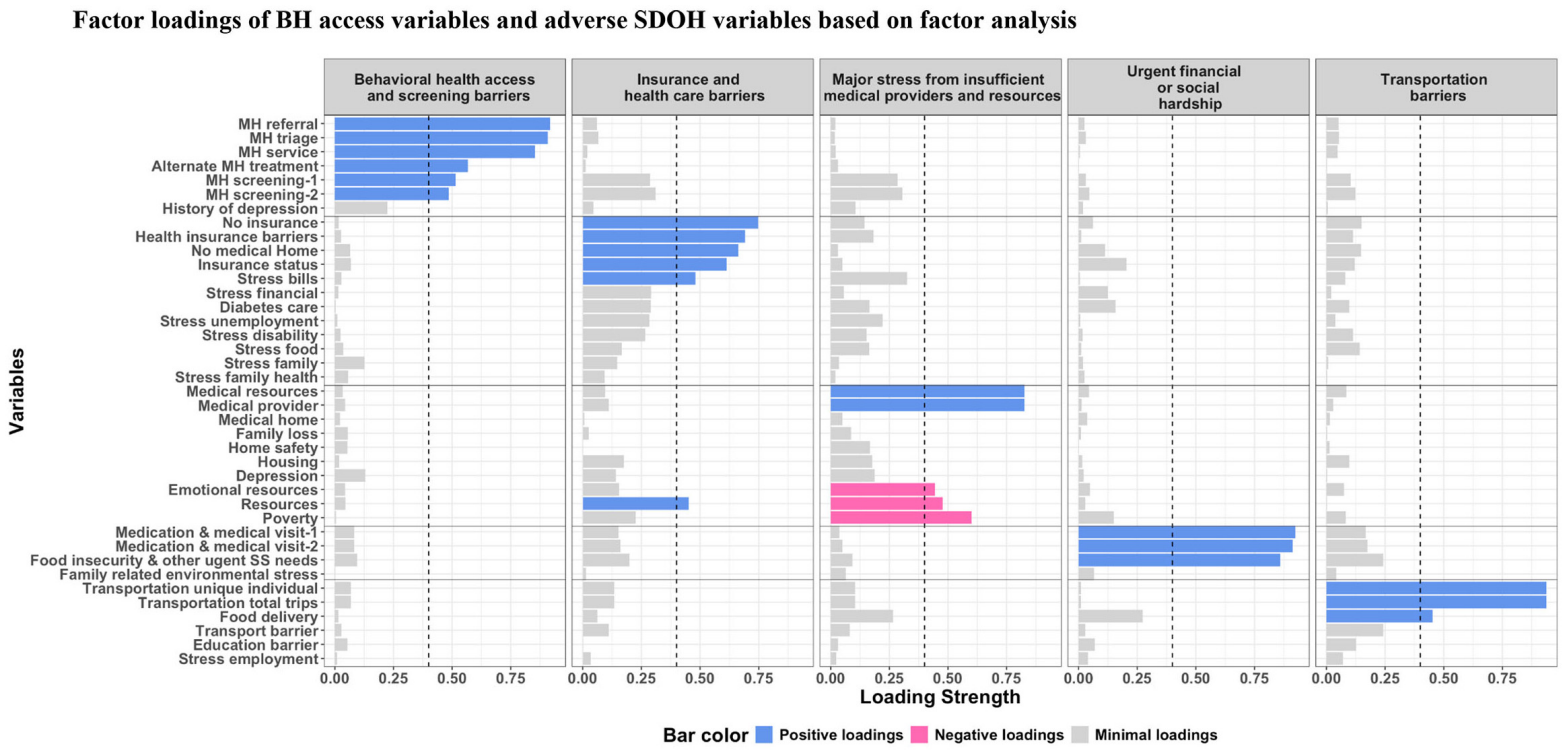
Factor loadings of behavioral health access and SDOH variables based on factor analysis.

We assessed the demographic and baseline characteristics of participants included in our analysis. Among *n* = 6,735 participants, 68.7% (*n* = 4,624) were female, and 69.5% (*n* = 4,652) preferred Spanish. Additionally, 57.8% (*n* = 3,665) were married, 33.4% (*n* = 1,830) were employed, 27.6% (*n* = 1,540) were overweight, while 64.3% (*n* = 3,592) were obese, and 44.5% (*n* = 2,995) had HbA1c > 10 at enrollment. We examined correlations among 39 SyV behavioral health access and SDOH variables. Some of these variables were strongly associated with each other (e.g., those related to referrals for mental health services at the region's mental health authority), so to handle collinearity, we conducted a factor analysis. We identified five factors, i.e., domains (one behavioral health access and four SDOH factors), which are no longer statistically significantly correlated with each other (Figure 1). The independent summary factor related to behavioral health was labeled as “Behavioral health access.” The four SDOH factors were labeled as: 1. Insurance and health care barriers; 2. Major stress from lack of medical provider and resources; 3. Urgent financial or social hardship; and 4. Transportation barriers. Factor patterns were clearly interpretable for the study population. The variables considered to be significant within each factor (i.e., variables with factor loading scores of 0.40 or higher) were all correlated to some degree except for lack of emotional resources, general lack of resources and being under the federal poverty level, which were negatively correlated.

Variables associated with depression exhibited high factor loading scores within Behavioral Health Factor 1, denoted as “Behavioral health access”. This factor is not a SDOH factor, but rather a screening and service access factor. SDOH Factor 1, labeled “Insurance and health care barriers,” prominently featured SDOH variables tied to insurance and medical bill obstacles, with substantial factor loadings. Moreover, SDOH Factor 2, identified as “Major stress from lack of medical provider and resources,” exhibited high positive loadings for SDOH variables related to the availability of medical providers and resources. Additionally, SDOH variables linked to food insecurity and other urgent financial and social service needs demonstrated elevated factor loadings within SDOH Factor 3, labeled “Urgent financial or social hardship,” a factor that also includes variables related to family violence, substance use disorder and safety issues. Furthermore, SDOH variables related to transportation displayed significant associations within SDOH Factor 4, labeled “Transportation barriers.” This factor also included educational barriers, a variable applied to participants with less than 8th-grade education, representing about half of our population. We chose an 8th-grade education as the cutoff because our population has, on average, fewer years of formal education than the general U.S. population (28). In addition, we performed K-modes clustering, and the main findings regarding factors remained very similar (Supplementary Table 2).

**Figure 2:**
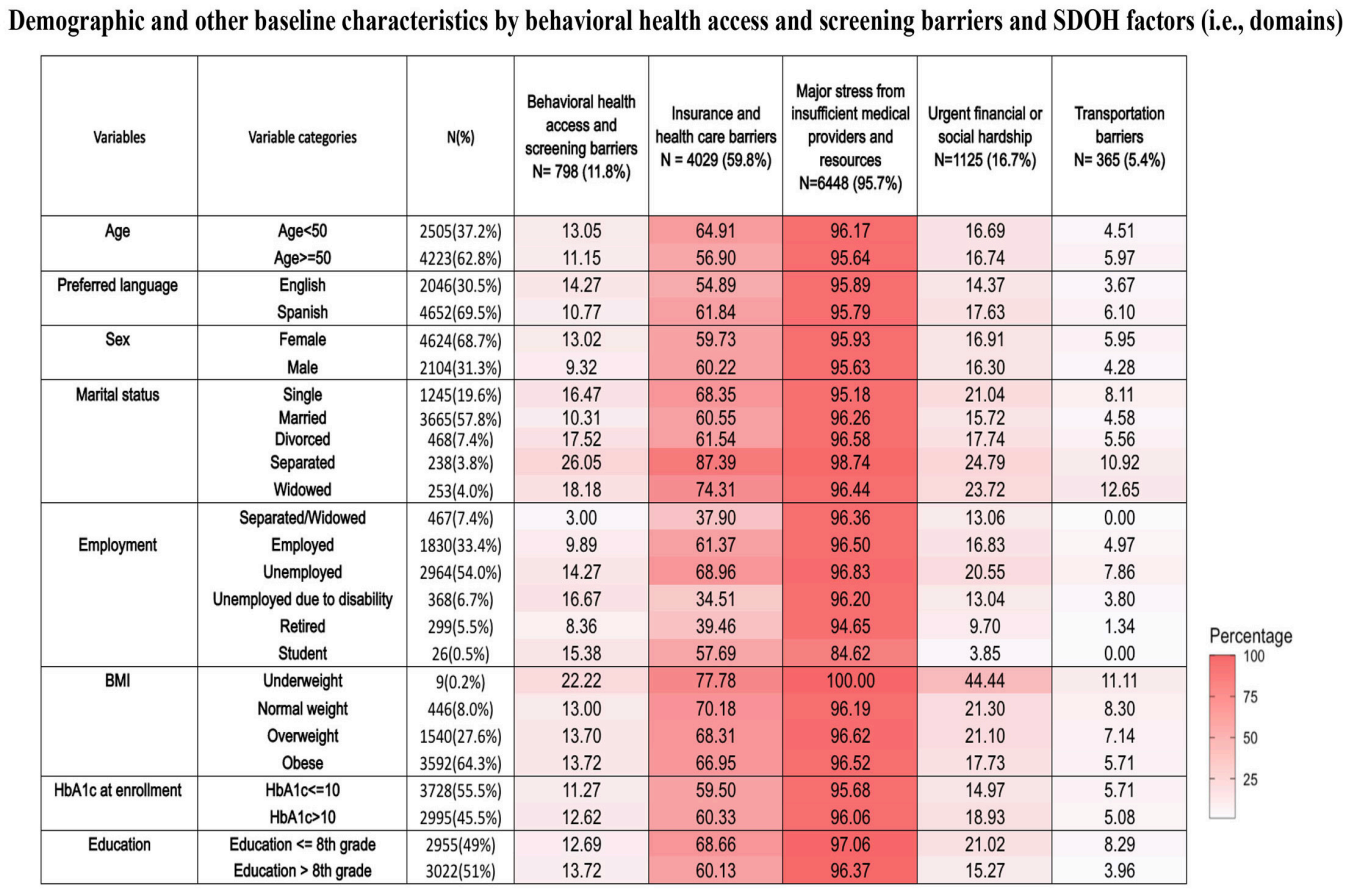
Demographic and other baseline characteristics by behavioral health access and SDOH factors (i.e., domains).

We further evaluated the associations of these factors with demographic and other baseline characteristics (Figure 2), including age, sex, marital status, preferred language, education level, employment status, and body mass index (BMI). We first quantified the percentage of participants who indicated at least one variable from each factor as their barrier/problem, presented using a color gradient in (Figure 2). Most participants had issues related to “Major stress from lack of medical providers and resources” (95.7%), and “Insurance and health care barriers” (59.8%). We then conducted univariable tests to assess the demographic/baseline characteristics associated with each of these 5 factors. We found different patterns over the categories of demographic/baseline characteristics, while no substantial differences were found in Major stress from lack of medical provider and resources. For example, compared to other categories of marital status, the ‘separated' or ‘widowed' group had more (i.e., darker color) participants who indicated “Insurance and health care barriers” and “Transportation barriers.” Males (compared to females) and participants who were employed or retired (compared to those who were unemployed) were less likely to have “Behavioral health access.” Compared to other employment groups, people who were retired or unemployed due to disability were less likely to have “Insurance and health care barriers.” Participants with underweight were more likely to have “Behavioral health access” and “Urgent financial or social hardship.” Compared to the higher educated group (8 years of education), the lower educated group had more participants who indicated “Insurance and health care barriers” (68.66 vs. 60.13%) “Urgent financial or social hardship” (21.02 vs. 15.27%), and “Transportation barriers” (8.29 vs. 3.96%).

Among 6735 participants, 258 (3.83%) had no high factor loadings on any factor, indicating the absence of severe issues. Moreover, 1161 (17.2%) had high loadings on one factor, 1941 (28.8%) on two factors, 2006 (29.8%) on three factors, and 1199 (17.8%) on four factors. Additionally, 170 (2.52%) participants displayed high loadings across all five factors, implying challenges across all five domains.

To evaluate the robustness of the identified factor structure for categorical variables, we also performed K-means clustering, an unsupervised clustering method suitable for binary and categorical data. The clustering results demonstrated substantial concordance with the factor analytic findings. Specifically, variables related to behavioral health access, insurance and healthcare barriers, urgent financial or social hardship, and transportation barriers clustered together similarly across both analytic approaches (Supplementary Table 2).

These findings suggest that the identified domains were stable and interpretable across distinct statistical methodologies. Despite this finding, the identified factors should be interpreted as exploratory latent domains intended to summarize correlated patterns of behavioral health access and SDOH needs, rather than strict psychometric constructs, as many observed variables were binary indicators derived from programmatic service records.

## Discussion

This study examined and characterized the salient factors among low-income, uninsured Latino individuals in a T2DM management program. Our results found one independent summary factor related to behavioral health access and four factors related to adverse social determinants: 1. Insurance and health care barriers; 2. Major stress from lack of medical provider and resources; 3. Urgent financial or social hardship; and 4. Transportation barriers.

Our unique sample was derived from an intervention effort to improve chronic disease management in a persistent poverty region on the Texas-Mexico border that has SDOH barriers, such as minimal education (38). In describing the complex and multifaceted SDOH needs of a low-income Latino population living with chronic conditions, specifically T2DM, we contribute to the existing literature on SDOH models, which were not specifically derived from a Latino population (2).

Our study is significant because prior work has largely examined individual SDOH factors in isolation, and there is limited consensus on the specific indicators needed to measure SDOH categories (39). As a result, measures of SDOH have often focused on single-need barriers, without considering the complexity of relationships between multiple SDOH needs (40). This study helps address the challenge of capturing overlapping and SDOH needs in individuals with type 2 diabetes and comorbidities. Generally, factor analysis provides a useful tool for examining and summarizing complex relationships among a large set of variables. In this study, variables were derived from a broad range of questions and interactions with participants that reflect their real needs. It explains the relationships among correlated variables by reducing them to a smaller number of conceptually meaningful composite variables. Our results group SDOH variables into factors and offer insights into how the variables and individual items relate. Our factor analysis results may help inform the measurement and prioritization of behavioral health access and SDOH needs in intervention settings.

Behavioral health access, or lack thereof, is salient for chronic disease management. While behavioral health access is not an SDOH, Latinos experience disproportionate barriers to care and cultural stigma about mental health, which create additional barriers to receiving that care (34) By better understanding the specific drivers and barriers for population groups underrepresented in the literature based on their lived experiences and needs collected from programmatic data, as was the case in the present study, disease management interventions can be strengthened (5, 25, 26).

The factor loadings provide insight into the unique needs of this population and identify which determinants should be the focus for measurement and intervention in this specific population. Certain factors, such as education, did not load significantly on “Transportation barriers,” which may be because of the overall low level of formal education found in the sample (49% of all participants had less than 8 years of education). Furthermore, education is highly correlated with other SDOH variables (e.g., employment, access to health insurance, income) included in this factor analysis, and thus these complex pairwise correlations could impact factor loadings. This analysis explored the relative strengths of variables and identified the underlying dimensions that explain the variance for this population. When interpreting factor loadings for the variables in each domain, complex pairwise correlations among those variables need to be considered. The factor loading scores should be interpreted as the relative strength of the weights, rather than as individual weights. “Insurance and health care barriers” and “Major stress from lack of medical provider and resources,” are not unexpected factors, considering the sample population. The Texas-Mexico border and specifically the region where the study took place, has some of the highest rates of poverty and uninsured, so access to healthcare is limited for much of the population (41, 42). Due to lack of insurance coverage and cost, the ability for patients with type 2 diabetes and comorbidities to pay for a doctor's visit and necessary medications is often out of reach, which impacts their ability to manage the disease. In Texas, where the study took place, health coverage is limited because the state did not expand Medicaid coverage, which is essential for low-income populations in the U.S. (43). Our sample has a large population born in Mexico and their migratory status could impact their ability to obtain insurance, qualify for benefits and earn sufficient income to cover the cost of healthcare.

### Limitations

Several limitations should be considered when interpreting these findings. First, the behavioral health access and SDOH variables were derived from programmatic case management and service-delivery records rather than from standardized, previously validated SDOH screening instruments. While these measures reflect real-world participant needs and service utilization within the intervention, their measurement properties have not been formally validated for research purposes and may differ from those used in other healthcare settings, potentially limiting comparability across studies. Documentation practices may also have varied across program staff over time, introducing potential inconsistencies in data collection. However, extensive quality control protocols were integrated into the chronic care management intervention which led to continuous data collection retraining. In addition, several measures used “check-all-that-apply” response formats, which may contribute to reporting inconsistencies and complex correlation structures among variables.

Secondly, missing data were relatively limited, but incomplete responses and variability in documentation may have influenced the observed correlation patterns and the resulting factor structure. Furthermore, many of the variables analyzed were binary or categorical indicators. While exploratory factor analysis has been widely applied in health services and survey research with large samples and involving categorical variables, it was originally developed for continuous variables and therefore has limitations when applied to binary measures. Consequently, the identified factors should be interpreted as exploratory latent domains that summarize correlated patterns of behavioral health access and SDOH needs rather than definitive psychometric constructs. To assess the robustness of these findings, we additionally performed K-modes clustering, an unsupervised learning approach designed specifically for categorical data, which yielded broadly similar grouping patterns.

Finally, analysis relied on baseline measures collected within a specific chronic disease management intervention serving predominantly low-income Latino participants living in two Texas-Mexico border counties. Therefore, the identified domains may reflect the unique social, healthcare access, and cultural context of this population and geographic region and may not generalize to other Latino populations, healthcare systems, or community settings with different demographic, socioeconomic, or healthcare characteristics. Future studies are needed to evaluate the reproducibility, external validity, and longitudinal stability of these domains in independent populations and settings.

## Conclusion

Overall, this study helps define useful domains of behavioral health access, screening barriers, and SDOH in a program database free of multicollinearity. This improves efficiency by reducing the number of variables needed to identify the most salient needs, with the potential to use the variables with the highest factor loadings in future analyses. The findings from our factor analysis can inform measurement for future interventions focused on multiple SDOH. This study contributes to a better understanding of behavioral health access and screening barriers, as well as SDOH access gaps, at the individual level among an at-risk Latino population, allowing for comparison with other settings and populations in future studies. Future studies could also build on this work to explore the association between the SDOH domains/factor groupings established in this study and other conditions and behaviors.

## Data Availability

The datasets used and/or analyzed during the current study are available from the corresponding author on reasonable request Lisa.mitchell-bennett@uth.tmc.edu.
