# Supplementary material for: Identifying salient behavioral health access and social determinant of health factors among a Latino population with uncontrolled diabetes living on the U.S.-Mexico border
Source: Front Public Health. 2026 Aug 5;14:1808640. doi: 10.3389/fpubh.2026.1808640 (PMC13486290; doi:10.3389/fpubh.2026.1808640)
Supplement: Supplementary file 1 [file Supplementary_file_1.docx]

**Supplementary Table 1. Programmatic Measures and Variables**

| Measure | Source (If Validated Tool) or Item Origin |
| --- | --- |
| Mental Health Severity measured by PHQ2/9 | Kroenke K, Spitzer RL, Williams JB. The Patient Health Questionnaire-2: Validity of a Two-Item Depression Screener. Medical Care. 2003; 41:1284-92.    Patient Health Questionnaire-2 (PHQ-2), a 2-item scale assessing depressive symptoms over the past two weeks. Participants respond on a 4-point Likert scale. |
| Self-report Previous Depression Diagnosis | Binary Yes/No when data missing from PHQ2 combined with this variable to create a binary depression variable |
| Behavioral Health Recommendation Referral (Community and Clinic Services) | BH Recommendation Survey (Enrollment & Follow-up)  Binary Yes/No |
| Behavioral Health Referral to Mental Health Authority | Behavioral Health Feedback Survey  Binary Yes/No |
| Compassion Fund Application Submitted for Review | Compassion Fund Application—Internal programmatic question—Binary Yes/No |
| No Medical Home | Internal programmatic question—Binary Yes/No |
| Transportation Referral | Internal programmatic question—Binary Yes/No |
| Social Work Referral | Internal programmatic question—Binary Yes/No |
| Resources/Services Provided | Internal programmatic question—Binary Yes/No |
| Food Insecurity | Internal programmatic question—Binary Yes/No |
| Self-reported “Major stressors in life” (answer list of stressors with unlimited answers):   - Financial - Financial resources for DM care - Bills - Employment related - General lack of resources - Lack of Medical Resources - Housing - Unable to work - Family in Mexico - Unemployed - Disability - Food - Home Safety - Depression - Family member's death - Family member's health - Family member's substance abuse - Family Health - No PCP - General Stress - General lack of resources - Justice system - Family distant | Questions adapted from Chronicle Diabetes Database Questions for Clinical Management of DM  Categorical (multi-select list of stressor categories)    Measure relates to the AADE-7 self-care behavior: Healthy coping |
| Transportation need | Documented as transportation referral by intervention personnel—Binary Yes/No |
| Emotional Resources | Questions adapted from Chronicle Diabetes Database Questions for Clinical Management of DM    Measure relates to the AADE-7 self-care behavior: Healthy coping  Numeric (scale score from validated questionnaire) |
| Barriers to care include:   - Transportation - Lack of emotional resources - Health Insurance | Internal programmatic question  Categorical (multi-select) |
| Stress Management Method (unlimited selection): | Questions adapted from Chronicle Diabetes Database Questions for Clinical Management of DM    Measure relates to the AADE-7 self-care behavior: Healthy coping  Categorical (multi-select) |

**Supplementary Table 2. Comparison of factor analysis domains and K-modes clustering results**

| **Factor Analysis Domain** | **Variables with Highest Factor Loadings (≥0.40)** | **Corresponding K-modes Cluster Pattern** | **Concordance Interpretation** |
| --- | --- | --- | --- |
| Behavioral health access and screening barriers | MH referral, MH triage, MH service, alternate MH treatment, MH screening-1, MH screening-2 | Behavioral health referral and screening variables clustered together consistently | High concordance |
| Insurance and health care barriers | No insurance, health insurance barriers, no medical home, insurance status, stress from bills | Insurance access and healthcare navigation variables grouped together | High concordance |
| Major stress from lack of medical provider and resources | Medical resources, medical provider, emotional resources, poverty | Resource limitation and provider-access variables clustered together | Moderate-to-high concordance |
| Urgent financial or social hardship | Medication & medical visit-1, medication & medical visit-2, food insecurity & other urgent social service needs | Financial hardship and urgent assistance variables grouped together | High concordance |
| Transportation barriers | Transportation unique individual, transportation total trips, food delivery | Transportation and mobility-related needs clustered together | High concordance |

**Supplementary Diagram 1. Analytic sample inclusion**

Number excluded n=0 This analysis included the entire sample of enrolled participants between 2013-2020

Final sample included in the analysis n=6735

Total Salud y Vida Participants enrolled in intervention during the study period (2013-2020) n=6735
